# A mobile health technology platform for quality assurance and quality improvement of malaria diagnosis by community health workers

**DOI:** 10.1371/journal.pone.0191968

**Published:** 2018-02-01

**Authors:** Jeremiah Laktabai, Alyssa Platt, Diana Menya, Elizabeth L. Turner, Daniel Aswa, Stephen Kinoti, Wendy Prudhomme O’Meara

**Affiliations:** 1 Department of Family Medicine, Moi University School of Medicine, Eldoret, Kenya; 2 Academic Model Providing Access to Healthcare (AMPATH), Eldoret, Kenya; 3 Duke Global Health Institute, Duke University, Durham, North Carolina, United States of America; 4 Department of Biostatistics and Bioinformatics, Duke University, Durham, North Carolina, United States of America; 5 Department of Epidemiology and Biostatistics, Moi University School of Public Health, College of Health Sciences, Eldoret, Kenya; 6 Fio Corporation, Toronto, Canada; 7 Division of Infectious Diseases and International Health, Duke University Medical Center, Durham, North Carolina, United States of America; Academic Medical Centre, NETHERLANDS

## Abstract

**Background:**

Community health workers (CHWs) play an important role in improving access to services in areas with limited health infrastructure or workforce. Supervision of CHWs by qualified health professionals is the main link between this lay workforce and the formal health system. The quality of services provided by lay health workers is dependent on adequate supportive supervision. It is however one of the weakest links in CHW programs due to logistical and resource constraints, especially in large scale programs. Interventions such as point of care testing using malaria rapid diagnostic tests (RDTs) require real time monitoring to ensure diagnostic accuracy. In this study, we evaluated the utility of a mobile health technology platform to remotely monitor malaria RDT (mRDT) testing by CHWs for quality improvement.

**Methods:**

As part of a large implementation trial involving mRDT testing by CHWs, we introduced the Fionet system composed of a mobile device (Deki Reader, DR) to assist in processing and automated interpretation of mRDTs, which connects to a cloud-based database which captures reports from the field in real time, displaying results in a custom dashboard of key performance indicators. A random sample of 100 CHWs were trained and provided with the Deki Readers and instructed to use it on 10 successive patients. The CHWs interpretation was compared with the Deki Reader’s automatic interpretation, with the errors in processing and interpreting the RDTs recorded. After the CHW entered their interpretation on the DR, the DR provided immediate, automated feedback and interpretation based on its reading of the same cassette. The study team monitored the CHW performance remotely and provided additional support.

**Results:**

A total of 1251 primary and 113 repeat tests were performed by the 97 CHWs who used the DR. 91.6% of the tests had agreement between the DR and the CHWs. There were 61 (4.9%) processing and 52 (4.2%) interpretation errors among the primary tests. There was a tendency towards lower odds of errors with increasing number and frequency of tests, though not statistically significant. Of the 62 tests that were repeated due to errors, 79% achieved concordance between the CHW and the DR. Satisfaction with the use of the DR by the CHWs was high.

**Conclusions:**

Use of innovative mHealth strategies for monitoring and quality control can ensure quality within a large scale implementation of community level testing by lay health workers.

## Introduction

Community-based health interventions deployed through Community Health Workers (CHWs) are becoming increasingly prevalent and important in resource-constrained settings [1, 2]. They can both extend the reach of health services into areas with limited access to facilities and reduce the burden on over-extended health systems by ‘task-shifting’ [3, 4]. Community case management for malaria (CCM) or home-based management of malaria is one such community intervention that was originally deployed in the mid-1990s as presumptive treatment of fevers with antimalarials at home [5]. It has evolved over the last two decades and the current best-practice for CCM couples malaria rapid diagnostic tests (RDTs) followed by artemisinin-combination therapy (ACTs) for those with a positive test [6]. Trained CHWs carry out both testing and drug dispensing within the community.

Several studies have shown that CCM can improve case management, and reduce hospitalization and mortality from malaria [7, 8, 9]. Although CHWs can correctly administer RDTs in the context of controlled research studies, their skill level is strongly correlated to the quality of training and the intensity of supervision and feedback [10]. This raises concerns about how to ensure high quality of diagnosis in large-scale programs where supervision may be limited, and routine quality assurance measures are not institutionalized. Large-scale implementation may benefit from new and innovative methods for monitoring the performance of CHWs using RDTs and ensuring patient safety.

**Fig 1:**
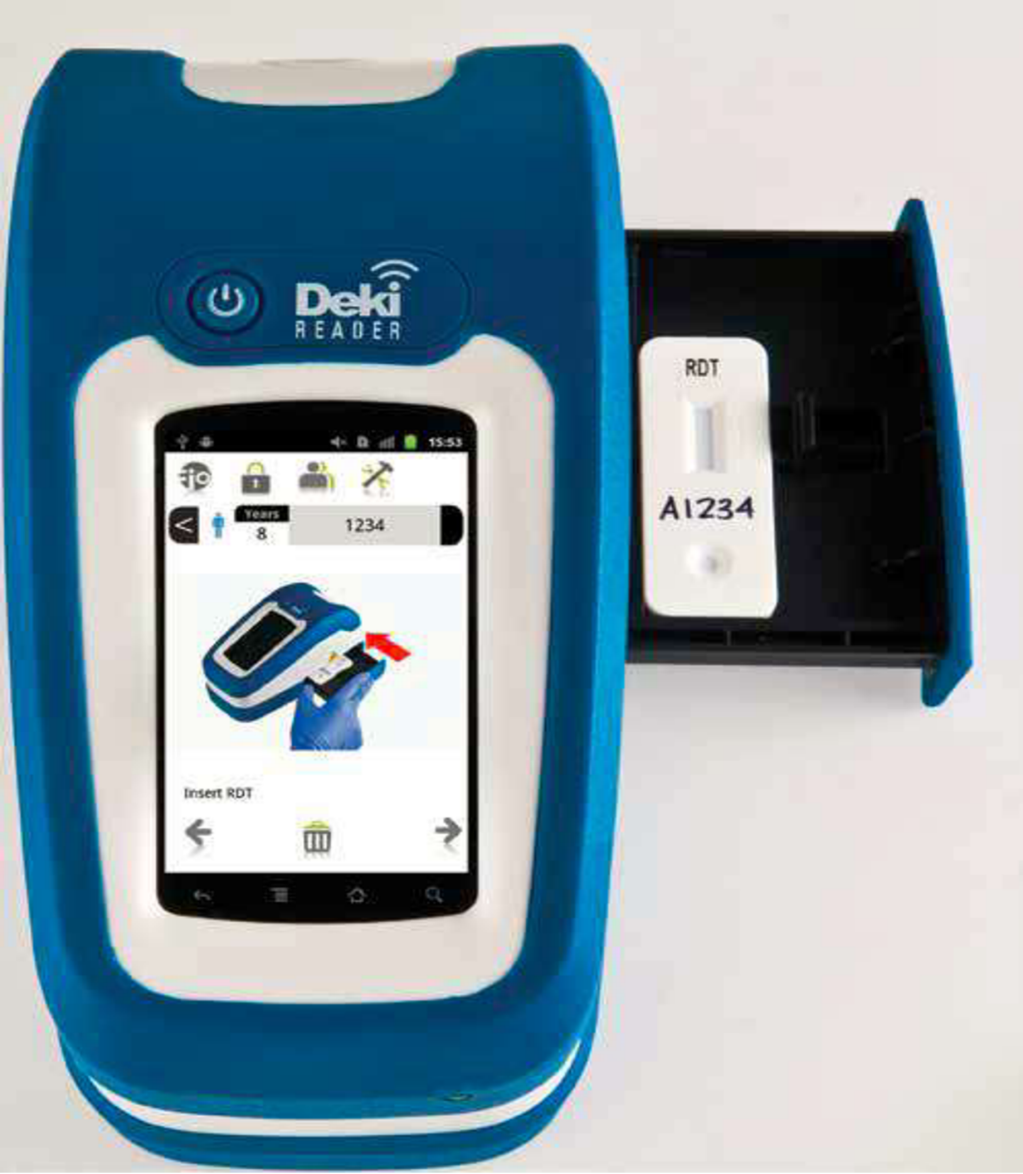
Deki Reader.

Mobile devices to improve regular communication and monitoring have been used in health facilities and been shown to increase efficiency and reduce costs [11]. In community-based interventions, mobile devices have been used for field data collection, health education and to receive reminders and alerts [12]. However, there is limited evidence for their role in monitoring skill performance. Our goal was to evaluate the use of a mobile device for monitoring and improving the quality of diagnostic services offered by community health workers as well as to objectively measure the performance of the CHWs. We tested an android-based platform called Fionet™, which consists of a mobile device, the Deki™Reader (DR) that interprets and provides results from RDTs and gives immediate feedback to the user on quality of the RDT procedure. The DR (Fig 1) then transmits all data, including a high-resolution image of the RDT to a cloud-based database in real time. In the secure portal, information from the field is displayed to show results in a custom dashboard of key performance indicators. The diagnostic performance of the DR has been proven to be comparable to visual interpretation [13, 14], with a sensitivity and specificity of 93.9% and 98.7% respectively for Plasmodium falciparum when compared to the gold standard [14]. We customized the device to allow the CHW to read and enter their interpretation first, before seeing the results from the device. This permitted real-time feedback and learning for the CHW as well as evaluation of CHW skill level. We deployed the device in a sample of CHWs participating in a large-scale diagnostic testing program in western Kenya that serves a population of more than 100,000 people.

We hypothesized that errors in performing RDTs and discordance between CHW and DR interpretation of RDTs would become less frequent as CHWs continued to receive real-time feedback from the DR. We also wished to identify any patterns in such errors that were associated with demographic characteristics and experience-level of the CHWs.

## Materials and methods

### Study population

This study was conducted within a large implementation trial of community-based malaria testing which is described in detail elsewhere. Briefly, two-hundred and seventy-one Community Health Workers were recruited and trained to perform malaria RDTs as part of a larger ongoing cluster-randomized trial conducted in three areas of western Kenya [15]. Participating CHWs were already established in their communities and previously trained to carry out basic health promotion and prevention activities according to the Ministry of Health curriculum. CHWs were given RDTs (Carestart Pf HRP2) and began testing of suspected malaria cases in their communities between July and October 2015. Basic demographic information about each CHW, as well as their previous training and experience with malaria testing, was collected on standardized forms at the time of initial training. The study team provided RDTs to the CHWs every 2–4 weeks, depending on usage. They were also given waterproof bags and were trained on correct storage conditions. Spot checks of RDT storage were made when the study team visited CHWs in the community.

In July 2015, we introduced the Fionet System [16] (from Fio Corporation) to remotely monitor the performance of a sample of CHWs as they processed RDTs in real time. Fionet System consists of two components: mobile devices (named Deki™Readers, DR) to assist in processing and automated interpretation of mRDTs; and an internet accessible cloud-based database which captures reports from the DRs in the field in real time. For a DR-evaluated RDT, the CHW wrote a unique study identifier on the RDT cassette, inserted it into the DR for the device to take a photograph, then removed the cassette and performed the RDT according to manufacturer instructions. After performing the test, the CHW inserted the cassette into the DR once again and recorded his or her reading of the test (i.e., positive, negative, or invalid). The DR took a second photograph of the final RDT for automated interpretation of the results. For our study, Deki Reader software was programmed to provide the automated RDT interpretation immediately after the CHW provided their interpretation, allowing for real-time feedback to the CHW performing the test. The DR automated interpretation could take the form of positive, negative, or invalid results. When the DR determined results were invalid, it also provided information about the source of the error, which could include RDT user errors such as too much blood, too little buffer, placing the sample in the wrong well, or reading the test after the prescribed time (20 minutes); or it could determine that the RDT itself was faulty (control line too low or unexpected line position). CHW interpretations, DR interpretations, and an image of the cassette were uploaded to a secure server and the study team reviewed results daily.

### Study procedures

100 CHWs were randomly selected in groups of 10 from the three study areas. They were required to give a verbal informed consent. Each group was trained to use the DR during a two-day, hands-on workshop. CHWs were asked to perform all of their tests with the DR and were instructed to repeat the RDT if the DR reported an error or if there was a discrepant reading result. They used the devices for a target of 10 tests. When all 10 CHWs in the group had reached at least ten tests, or the time with the DR exceeded 44 days, the DRs were rotated to the next group of 10. The CHWs had contact information for both the study team and the Fionet technical support team and they were encouraged to contact either when they experienced any problems using the DR. In addition, every CHW was actively followed up by phone or in person at least once, and more frequently if the observed testing rate was low, to ensure that any problems with the device were resolved. Before rotating the DR to a new group of CHWs, the study team confirmed they were in good working order. The DRs were maintained according to the manufacturer’s instructions.

The error and agreement rates were monitored daily from the cloud database and communicated to the supervision team. Those who had errors received individual on-the-job training from the study team. A subset of the participating CHWs was interviewed about their experiences in the malaria testing program. Questions were designed to elicit their perceptions of the ease of use, usefulness, and desire to use the DR in future.

### Data analysis

For the purposes of analysis, we categorized RDT performance errors into two types: processing errors and reading errors.

We defined a *processing error* as an error by the CHW in preparation of the RDT. These included too much blood, too little buffer, placing the sample in the wrong well or reading the test after the prescribed time (20 minutes). Observations that included errors that occurred as a result of a faulty RDT were excluded from the processing error analysis since such errors arose from product defects and not due to any error made by the CHW.

We defined a *reading error* as any disagreement between the CHW and the DR in interpretation of the RDT. In the case of invalid results, we did not remove observations where the DR determined that the RDT was faulty (as was done in analysis of processing errors) since our main concern was that a CHW recognize that the RDT results were invalid, no matter the source of the error. Defective cassettes, for example those where a control line failed to appear, should be read by the CHW as ‘invalid’.

To assess the representativeness of our random selection of 100 CHWs from the larger study population of 271 CHWs, we compared demographics (gender, age, and education) between the subsample of 100 CHWs selected for the DR and the 117 not selected for the DR sample. We performed chi-square tests for categorical variables (gender and education) and two-sample t-tests for age to test hypotheses that the CHWs we selected did not differ significantly from those not selected.

We computed summary statistics across CHWs to describe the sample of tests performed by each CHW including: total tests performed, whether the target of at least 10 tests was reached, the fraction of total tests on which Deki was used (during the Deki study period), the number of days the CHW had the DR, mean time between tests, number RDTs performed prior to the Deki study, and number of any processing errors or any reading errors. Categorical variables were summarized with frequencies and percentages, normally distributed continuous variables with means and standard deviations, and continuous variables with skewed distributions with medians and the IQR (reported as the 25^th^ and 75^th^ percentiles).

Interpretations of RDTs made by the CHW versus the DR are summarized in cross-tabulation to illustrate frequency of non-concordance of various types. Invalid results are separated into two columns to denote frequency of processing errors (errors made by CHWs) versus invalid errors resulting from faulty RDT cassettes.

Regression modeling was used to explore the relationship between CHW characteristics and odds of processing and reading errors. The binary outcome measures of interest were incorrect processing of the RDT (Model 1) and incorrect interpretation of the RDT (Model 2). RDTs performed prior to the DR Deki study could not be determined for six participants. Due to the small sample size, we wanted to ensure such missing data did not substantially affect the other parameter estimates, therefore, we present regression results with and without the covariate for both outcomes, yielding four total regressions. We used logistic regression fit with generalized estimating equations to account for clustering due to repeated RDT reads by the same CHW and assumed an independence working correlation matrix. Descriptive summaries showed that the number of tests performed by the CHWs varied widely and was potentially correlated to CHW characteristics, making informative cluster size (ICS) a likely characteristic of the data [17]. We corrected for ICS by including the total number of tests performed by the CHW using the DR (i.e. the cluster size) as a fixed effect in the model (S1 Text) [18]. Coefficients from the regression models were exponentiated to obtain odds ratios (OR) relating characteristics of the CHWs to the odds of the outcome. Explanatory variables of interest included age and education-level of the CHW, previous experience with RDTs as measured by the number of tests conducted before using the DR, experience with the DR as measured by the position of the test (in quartiles) in the sequence of tests performed by the CHW using the DR, time since the previous test was performed, and the percent of total tests performed during the period that the DR was used. Continuous explanatory variables were standardized to preserve interpretability of the intercept term.

## Results

### Study population

One hundred CHWs were trained to use the DR between July 2015 and April 2016. The median age was 42 years (IQR: 37.4–48.2) and the majority were female (64%). A majority (64%) completed secondary school or above (Table 1). The subset of CHWs participating in the study reflect the overall demographic composition of the trained CHWs in age, gender, and education level. Three CHWs did not perform any RDTs with the Deki. The remaining 97 completed between 1 and 48 tests (median = 12, IQR: 8 – 17) (Table 2). Those CHW who used the DR at least once performed a median of 19 RDTs (IQR: 7–40) prior to using the DR. CHWs held the DR for a median of 17 days (IQR: 10.9 – 23.3). Of the CHWs who performed any tests with the DR, most CHWs (66%) met or exceeded the required 10 tests. On average, tests were not performed every day, with a median amount of time between tests at 1.7 days (IQR: 1.1, 2.7). Almost a third (28.9%) of participants made at least one processing error, while half (52.6%) made at least one reading error.

**Table 1:** CHW demographic characteristics^1^.

(N = 100)	
Gender	
Female *n (%)*	64 (64.0%)
Age (years)^2^ *median (IQR)*	42.1 (37.4, 48.2)
Education category (2-level)	
None or completed primary only *n (%)*	36 (36.0%)
Completed secondary *n (%)*	64 (64.0%)

^1^Categorical variables are expressed as N (%), continuous as median with interquartile range (IQR)

^2^Age data available for (N = 96) participants

**Table 2:** Deki Reader use.

N = 97	
Days used Deki Reader *median (IQR)*	17.0 (10.9, 23.3)
Total tests during Deki Reader study period *median (IQR)*	14.0 (10.0, 25.0)
Total tests with Deki Reader (excluding repeats) *median (IQR)*	12.0 (8.0, 17.0)
Number of Deki Reader tests performed per day^2^ *median (IQR)*	0.6 (0.4, 0.9)
Performed at least 10 tests with Deki (excluding repeats) *n(%)*	64 (66.0%)
Time between tests (mean days) *median (IQR)*	1.7 (1.1, 2.7)
RDTs performed before Deki Reader study *median (IQR)*	19.0 (7.0, 40.0)
Any processing error^3^ *n(%)*	28 (28.9%)
Any reading error^4^ *n(%)*	51 (52.6%)

^1^Includes only CHWs who performed at least one test

^2^Tests-per day during the total time CHW had the Deki Reader

^3^ Processing Error is any error caused by the user of the Deki Reader. Processing errors include: "RDT was placed in the device too late for analysis", "Smeared RDT is Unreadable", "Too much blood", and "Blood in blood well", this does not include faulty RDTs. This variable is CHW level and indicates the presence of at least one processing error

^4^Reading Error is any non-concordant result between CHW and Deki. Reader. Processing errors and faulty RDTs are included and considered correctly read if CHW identifies them as invalid. This variable is CHW level and indicates the presence of at least one reading error.

### Outcome of testing and errors detected

In total, 1,251 primary tests and 113 repeat tests (following a processing error or reading discrepancy) were completed with the DR over the course of the study. Overall, 91.6% of tests were performed correctly with agreement in interpretation between the DR and CHW (Table 3). Out of the 1251 primary tests, 17 (1.4%) had an error resulting from a faulty RDT cassette, while 44 (3.5%) had a processing error. Of the 61 (4.9%) invalid results, 9 (14.8%) were correctly identified as being invalid by the CHW. The most common user processing error was placing the RDT cassette into the DR after the 20-minute waiting time had elapsed (N = 21, 34% of processing error total) (Table 4). The device was programmed such that it was not possible to read the cassette too early, therefore timing errors only occurred if the cassette was placed into the device after 22 minutes (the maximum time-limit for reading the cassette). When 20 minutes had elapsed, the DR sounded a loud reminder alarm alerting the user to insert the cassette. Failure to respond to this alarm could only indicate that the CHW had left the device unattended and could not hear the alarm, or they struggled with the correct usage of the DR and did not understand what the alarm meant. The latter scenario is a technical challenge, but not necessarily a CHW error that might compromise quality. This is supported by the observation that the image of the cassette in >90% of instances of this error showed that no sample or buffer had been loaded, indicating that the CHW likely misunderstood when to insert the blank, unused cassette and when to initiate the test.

**Table 3:** Interpretation concordance between CHW and Deki Reader.

	Deki Reader Result*				
CHW Interpretation of RDT	Faulty RDT^1^	Processing Error^2^	Negative	Positive	Total
Invalid^3^	8	1	10	6	25
Negative	5	32	934	26	997
Positive	4	11	10	204	229
**Total**	17	44	954	236	1251

*Concordant results highlighted in gray

^1^Faulty RDT is an error originating from a faulty RDT cassette, not user error. DR interpretation includes "control line too low" and "unexpected line position, cassette may be faulty"

^2^Processing Error is any error caused by the person preparing the RDT. Processing errors include: "RDT was placed in the device too late for analysis", "Smeared RDT is Unreadable", "Too much blood", and "Blood in blood well"

^3^Invalid as interpreted by the CHW prior to Deki Reader feedback, result could be invalid due to a faulty RDT or a processing error

**Table 4:** Frequency and type of RDT device and processing errors reported by Deki Reader.

Characteristics of Invalid RDT Results		
	N	(%)
**Faulty RDT**^1^ **cassette (N = 17)**		
Control line too low	17	28%
**Processing Error**^2^ **(N = 44)**		
Blood in blood well	2	3%
RDT too late for analysis	21	34%
Smeared RDT is Unreadable	13	21%
Too Much Blood	8	13%
**Total**	61	100%

^1^Faulty RDT is an error originating from a faulty RDT cassette, not user error. DR interpretation includes "control line too low" and "unexpected line position, cassette may be faulty"

^2^Processing Error is any error caused by the person preparing the RDT. Processing errors include: "RDT was placed in the device too late for analysis", "Smeared RDT is Unreadable", "Too much blood", and "Blood in blood well". ‘Smeared RDT is unreadable’ could arise from too much blood or inadequate buffer.

Among all primary tests, discordant readings were recorded in 25 out of 229 (10.9%) positive CHW readings, 63 out of 997 (6.3%) negative readings, and 16 out of 25 (64%) invalid readings. Of all of the tests that either had a processing error (N = 61) or a discordance between the CHW interpretation and the DR reading (N = 52), 62 (54.9%) were repeated, and 49 (79%) of these achieved a valid, concordant result upon repeat. The study team noted on review of the photographs that a positive interpretation was not possible by the naked eye for 13 tests where the DR returned a positive interpretation and the CHW entered a negative interpretation. Seven of the 13 were repeated, four of which had a processing error and three of which returned a negative result by the DR on the second test.

### Distribution of errors

**Fig 2:**
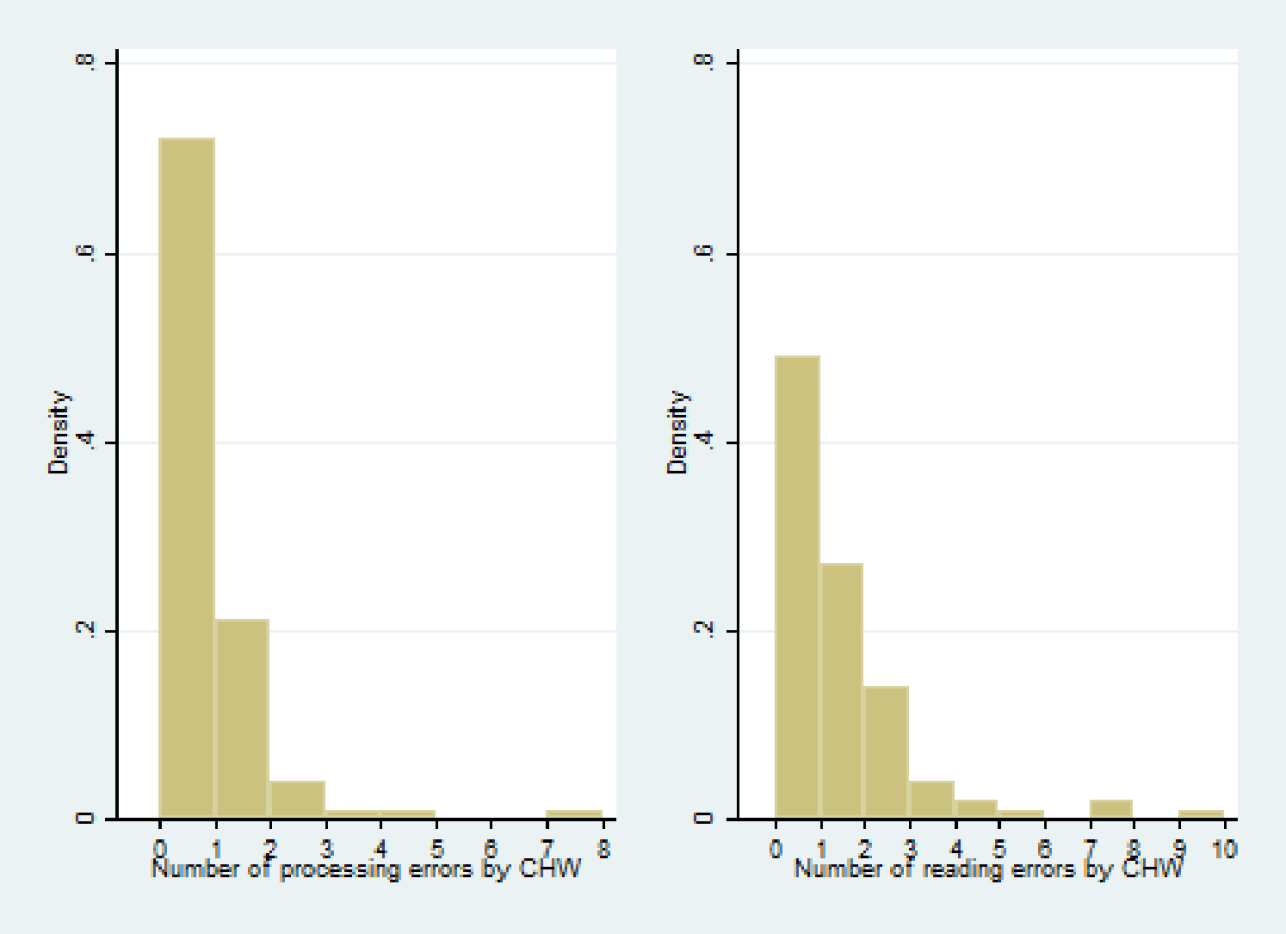
Distribution of processing errors (N = 44) and reading errors (N = 104) by CHWs (N = 97).

Overall, there were few errors, but slightly more than half (51.5%) of CHWs performed at least one error, with 29.6% having at least one processing error and 51.5% at least one reading error. The distribution of errors was highly skewed (Fig 2). One CHW was responsible for 18% of the 61 processing errors, 21 CHWs made only a single processing error and 69 CHWs made no processing errors. Likewise, 41 CHWs (41.8%) made only one or two reading errors and the remaining 49 errors were attributable to 13 CHWs.

### Multivariable analysis

We analyzed the outcome of each of the 1,251 primary tests to understand the role of previous experience, CHW demographics, and real-time learning from the DR in correct execution and interpretation of the RDTs.

We examined risk factors associated with the outcome of *processing errors* (Table 5). We did not find evidence that CHW-level characteristics of age, education, and previous experience performing RDTs (as measured by number of tests prior to the Deki study) was correlated with the odds of making a processing error. Likewise, test-level characteristics did not correlate with the odds of making processing errors. Taken together, this suggests that processing errors were both rare and relatively random. Although not independently and significantly correlated with the odds of an error, there is a notable trend towards lower odds of a processing error with increasing quartile of test order number. In other words, there is some evidence that the odds of a processing error may become lower as the number of tests executed with the DR increased, although the confidence intervals for these estimates were wide.

**Table 5:** Test-level regressions^1^ of association of processing and reading errors from Deki Reader with CHW and test characteristics.

	Processing Errors^2^ (N = 1,234)		Reading Error^3^ (N = 1,251)	
Variables	Odds Ratio	Odds Ratio	Odds Ratio	Odds Ratio
**CHW Characteristics**				
Age (standardized)	1.05	1.07	1.03	0.92
	(0.73–1.52)	(0.70–1.61)	(0.81–1.30)	(0.71–1.19)
Education category (2-level) = 1, Completed secondary	1.68	1.68	1.38	1.20
	(0.81–3.45)	(0.72–3.90)	(0.87–2.18)	(0.72–2.01)
Tests prior to Deki (standardized^4^^,^^5^)		1.06		0.92
		(0.77–1.48)		(0.73–1.15)
**Test Characteristics**				
Quartiles of sequence number = 2, 2nd quartile	1.15	1.10	0.86	0.93
	(0.40–3.32)	(0.38–3.19)	(0.45–1.65)	(0.48–1.83)
Quartiles of sequence number = 3, 3rd quartile	1.09	0.94	0.88	0.93
	(0.37–3.22)	(0.31–2.82)	(0.45–1.71)	(0.47–1.86)
Quartiles of sequence number = 4, 4th quartile	0.71	0.56	0.84	0.86
	(0.22–2.24)	(0.17–1.83)	(0.41–1.73)	(0.41–1.84)
Days from last test	1.07	1.07	**1.12**	**1.11**
	(0.98–1.16)	(0.97–1.17)	**(1.07–1.18)**	**(1.04–1.17)**
Observations	1,231	1,155	1,248	1,172
Number of CHWs	96	90	96	90
Constant (i.e. intercept)^6^	0.01	0.01	0.04	0.05
	(0.00–0.03)	(0.00–0.03)	(0.02–0.08)	(0.02–0.10)

^1^Generalized Estimating Equations clustered at CHW-level, with independence working correlation, adjusted for cluster size and proportion of RDTs for which Deki Reader was used, coefficients exponentiated for odds ratio interpretation

^2^Processing errors (N = 44) exclude errors resulting from faulty RDTs (N = 17)

^3^Reading error is any non-concordant result between CHW and Deki. Processing errors (N = 44) and faulty RDTs (N = 17) are both included and considered correctly read if CHW identifies them as invalid

^4^Continous variables standardized by mean centering and dividing by sample standard deviation

^5^ The number of tests conducted before using the Deki Reader could not be determined for six participants

^6^Constant term is expressed in odds

CHW = Community Health Worker

When examining reading errors, we did not find evidence that CHW-level characteristics were associated with odds of making a reading error (Table 5). Among test-specific characteristics, an increase of one day from the last test performed was associated with an 11% increase in odds of reading error (95% CI: 4% - 17%) suggesting that among CHWs who performed similar numbers of tests and used the DR a similar fraction of the time, more recent testing and feedback from the DR was positively associated with correct test interpretation.

### Operational challenges and CHW perspective

We documented some operational challenges using the DR. First, keeping the DR charged required some organization and forethought. Second, network availability in many of the communities was poor which delayed appearance of the results in the portal and also made technical support challenging. During the training and implementation, we noted that many CHWs had difficulty with the touchscreen technology and the complicated user interface. A large proportion of the CHWs did not use the DR consistently with each and every client during the time they had the DR; forty percent used the DR for less than 75% of the RDTs they conducted during the time they held the DR.

Despite the challenges perceived by the study team, CHWs reported positive experiences using the DR. During interviews with 30 randomly sampled DR users, 87% indicated that they would like to use the DR as part of their routine work and 94% said the DR helped improve their ability to perform RDTs correctly. A slightly lower percent indicated the DR was easy to use correctly (65%) which agreed with the processing errors observed.

## Discussion

The role of CHWs in achieving high coverage of health care interventions, particularly in resource-constrained areas, cannot be overemphasized. The challenges faced by CHWs in taking on more diverse and medicalized roles can be mitigated by effective and regular supervision, which has also been linked to CHW motivation [10, 19]. Yet supervision has been noted as one of the most challenging aspects in implementing CHW programs [20, 21]. Constraints including geographical, economic and limited human resources which all impact negatively on the quality and coverage of supervision and monitoring [22]. Even where regular and frequent supervision is possible, monitoring an intervention such as mRDT testing where direct observation and immediate feedback is desirable for diagnostic accuracy would be untenable in large-scale programs. In this study, use of an innovative mHealth strategy designed to simultaneously remotely monitor and provide real-time feedback to CHWs using malaria RDTs was explored.

The CHWs performed over 96% of the tests correctly and interpreted more than 92% correctly (including invalid cassettes). The largest number of reading errors was for invalid tests. However, when photographs were reviewed, most of the cassettes designated invalid by the CHW were in fact readable and valid, indicating more emphasis should be placed on training CHWs how to identify cassettes which cannot give valid results, either from a manufacturing defect or poorly prepared tests. The DR did not identify any cassettes that were missing a control line. The relatively few errors observed confirms that RDTs can be performed by CHWs and other lay workers with appropriate training and supervision [23, 24]. The error rates are comparable to those reported in health facilities among trained health workers [25], and among CHWs under direct observation [24]. The errors were highly overdispersed; a small group of CHWs was responsible for the majority of errors. However, characteristics such as age, education and experience were not correlated with the odds of making either a processing or reading error. This suggests that a device such as the Deki Reader could be very useful in identifying this group of CHWs with higher error rates. They could then be followed up with more intensive supervision and on-the-job training. Enabling targeted supervision could greatly enhance the effectiveness of supervision within the limited resources of a program.

The frequency and number of tests performed with the Deki Reader were related to the odds of committing and error. There was a tendency towards lower processing error rates with increasing number of tests performed although did not reach statistical significance. This could be a result of real-time feedback on errors or possibly increased familiarity with the device over time. The number of reading errors increased with an increase in the period between tests, suggesting that regular practice helps to maintain skill-level and accuracy.

There are several limitations that may affect the results. First, the number of errors in RDT performance was small, making it difficult to make inference on associated CHW and test-level characteristics. Second, the DR was not optimized for lay persons and some CHWs experienced challenges with the device. These people were likely to not have performed many or any tests with the DR and are likely to be under-represented in this analysis. For example, this may be reflected in the large number of errors like ‘placed in device too late for analysis’ or the insertion of a blank cassette at the end of the 20 minutes. We performed diagnostics and adjusted our regression models to account for informative cluster size, however, there may be unmeasured factors that would help explain differences in cluster size and allow us to more accurately identify characteristics associated with errors. For this reason, future approaches should begin with user-centered design. Third, the quality of the RDTs may be affected by storage and transportation conditions. In as much as spot checks of RDT storage by the CHWs were done, not all CHWs were evaluated for optimal storage, and this may affect the quality of the results. CHW programs should include collecting quality control samples from CHWs to ensure the tests are stored properly and quality is maintained. Finally, some cassettes that were interpreted as positive by the DR could not be read as positive by the naked eye. When repeated, they were often negative at the second test. This indicates need for continued calibration of the device to avoid confusing and potentially discouraging the CHWs. Despite these limitations, the study demonstrates that use of mobile technology as a tool for monitoring performance and quality improvement is feasible and can be explored in large-scale programs.

The major advantage of the Deki Reader was the opportunity to monitor in real-time the diagnostic quality and accuracy of RDTs performed by CHWs. The device could interpret and provide automated feedback on RDT preparation and interpretation in real time without being connected to the cellular network, which allows CHWs to receive feedback and understand their errors in real-time. Photographs captured during the test procedure allowed supervisors to review results daily. It is impossible to accomplish this with routine supervision unless the CHW attends to a patient in the presence of the supervisor. Even reviewing used cassettes at regular meetings cannot capture errors such as the timing of interpretation, mishandling of the blood sample, or inadequate buffer. This is the first report of which we are aware that evaluates the quality of CHW diagnosis in their routine workflow, outside of a training or supervision setting.

## Conclusion

Use of innovative mHealth strategies for monitoring and quality control can enhance quality, help target supervision, and ensure diagnostic accuracy within a large-scale implementation of community level testing by lay health workers, thus overcoming the barriers associated with traditional supervision methods.

## Supporting information

S1 Text Patterns of heterogeneity in error rates. (DOCX)
