# Supplementary material for: A mobile health technology platform for quality assurance and quality improvement of malaria diagnosis by community health workers
Source: PLoS One. 2018 Feb 1;13(2):e0191968. doi: 10.1371/journal.pone.0191968 (PMC5794091; doi:10.1371/journal.pone.0191968)
Supplement: S1 Text — (DOCX) [file pone.0191968.s001.docx]

**Supplementary file**

We identified patterns of heterogeneity in error rates by cluster size descriptively (Supplementary Table 1), noting that higher error rates were apparent in those with the lowest and highest number of Deki Reader (DR) tests. We also noted that the DR was used a substantially lower proportion of the time among those community health workers (CHWs) with relatively few observations. Due to a relatively low percentage of both processing and reading errors, we used zero-inflated Poisson regressions^26^ (ZIP) models to test the hypothesis that informative cluster size (ICS) was present in our data. We specified the ZIP models using the number of tests as a covariate in the count model and the inflation equation and the rate of processing and reading errors as the dependent variable. The ZIP models revealed that cluster size was predictive of the count of processing errors (p<0.001) and reading errors (p<0.001) for CHWs with a non-zero count of errors, though cluster size did not inform the binary probability of having a positive count of errors. ICS can be potentially corrected by including cluster size as a covariate in the model, if the CHW level characteristics are balanced by cluster size and cluster size in not on the causal pathway between CHW characteristics and probability of error.^2^ In order to test the hypothesis that such characteristics were independent of clusters size, we performed Wilcoxon rank sum tests for education variables and calculated spearman rank correlation for age and number of previous tests. We did not find strong evidence of dependence between number of tests and age (ρ=-0.18), education (p=0.30), or number of previous RDTs (ρ=0.23), indicating that controlling for cluster size as a fixed effect was an adequate approach for correcting the effects of ICS on our regression analyses. In addition, due to notable patterns seen in the proportion of RDTs for which DR was used, this was included as an additional control to further alleviate the effects of selection bias.

**Supplementary Table 1.** Error rates and fractions of tests performed with Deki Reader by total Deki tests performed

| **Quartile for total tests performed with Deki Reader** | **Number of CHWs** | **Total Deki tests** | **Total tests** | **% of tests for which DR was used** | **% of tests with processing error** | **% of tests with reading error** |
| --- | --- | --- | --- | --- | --- | --- |
| 0 to 7 | 27 | 97 | 238 | 40.8 | 2.1 | 8.2 |
| 8 to 12 | 30 | 313 | 383 | 81.7 | 1.9 | 6.7 |
| 13 to 16 | 18 | 258 | 267 | 96.6 | 2.7 | 5.8 |
| 16-48 | 25 | 583 | 838 | 69.6 | 5.0 | 10.3 |
| Total | 100 | 1251 | 1726 | 72.5 | 3.5 | 8.3 |
